# Identifying *HIF1A* and *HGF* as two hub genes in aortic dissection and function analysis by integrating RNA sequencing and single-cell RNA sequencing data

**DOI:** 10.3389/fcvm.2024.1475991

**Published:** 2024-10-16

**Authors:** Hai-Bing Li, Chang Liu, Xiang-Di Mao, Shu-Zheng Yuan, Li Li, Xin Cong

**Affiliations:** Department of Physiology and Pathophysiology, School of Basic Medical Sciences, Peking University Health Science Center, State Key Laboratory of Vascular Homeostasis and Remodeling, Beijing, China

**Keywords:** aortic dissection, RNA-sequencing, single-cell RNA sequencing, weighted gene co-expression network analysis, protein–protein interaction network, hypoxia-inducible factor 1 subunit alpha, hepatocyte growth factor

## Abstract

**Objective:**

Aortic dissection (AD) is a severe aortic disease with high mortality, and its pathogenesis remains elusive. To explore the regulatory mechanisms of AD, we integrated public RNA sequencing (RNA-seq) and single-cell RNA sequencing (scRNA-seq) datasets to screen the hub genes of AD and further analyzed their functions, which may provide references to the diagnosis and treatment of AD.

**Methods:**

Four AD-related datasets were obtained from the Gene Expression Omnibus (GEO) database. Weighted gene co-expression network analysis and differential expression analysis were applied to identify overlapping genes in dataset GSE153434. Protein–protein interaction (PPI) network was constructed based on overlapping genes. Five methods (closeness, degree, EPC, MCC, and MNN) were used to pick hub genes. The receiver operating characteristic curve was used to evaluate the diagnostic efficiency of the hub genes in extra datasets GSE98770 and GSE52093. scRNA-seq dataset GSE213740 was used to explore the expression and function of the hub genes at the single-cell level. Quantitative real-time polymerase chain reaction was used to verify the expression of hub genes in beta-aminopropionitrile (BAPN)-induced mouse thoracic aortic aneurysm and dissection (TAAD) model.

**Results:**

A total of 71 overlapping genes were screened by intersecting the significant genes in the pink module and the differentially expressed genes. A PPI network with 45 nodes and 74 edges was generated, and five top hub genes (*HIF1A*, *HGF*, *HMOX1*, *ITGA5*, and *ITGB3*) were identified. All the hub genes had area under the curve values above 0.55. scRNA-seq data analysis showed that *HIF1A* was significantly upregulated in macrophages and *HGF* was significantly upregulated in vascular smooth muscle cells (SMCs) of the ascending aortas in AD patients. HIF1A may transcriptionally regulate multiple downstream target genes involving inflammation (*TLR2*, *ALOX5AP*, and *MIF*), glycolysis (*ENO1*, *LDHA*, and *GAPDH*), tissue remodeling (*PLAU*), and angiogenesis (*SERPIN* and *VEGFA*). HGF may participate in the signaling among SMCs, fibroblasts, and endothelial cells through binding to different receptors (MET, EGFR, IGF1R, and KDR). The mRNA expression of *Hif1a*, *Hgf*, and their target genes, including *Alox5ap*, *Serpine1*, *Tlr2*, *Plau*, *Egfr*, and *Igf1r*, was significantly upregulated in aortic tissues of BAPN-treated mice.

**Conclusion:**

By integrating RNA-seq and scRNA-seq data, we identified *HIF1A* and *HGF* as two hub genes with good diagnostic efficiency for AD. HIF1A in macrophages may promote AD formation by promoting inflammation, glycolysis, tissue remodeling, and angiogenesis, and HGF may mediate signaling among SMCs, fibroblasts, and endothelial cells in the development of AD.

## 1 Introduction

Aortic dissection (AD) is a lethal vascular condition with a rapid onset, difficult diagnosis, and high mortality (1, 2). The main pathological characteristics include the destruction of the intima and the formation of true and false lumens. AD can be caused by hereditary or metabolic factors. Hereditary AD is caused by genetic defects such as Marfan syndrome (FBN1 defect). Metabolic AD is mostly secondary to hypertension and atherosclerosis (3). The treatment for acute AD is complicated, and surgery is still the only therapy at present (4, 5). Aortic smooth muscle cell (SMC) loss, extracellular matrix degradation, and inflammatory response are generally involved in the development of AD (6). Although many advancements have been achieved in the pathogenesis of AD, no reliable drugs are available to slow down AD formation. Therefore, it is critical to understand the molecular basis of AD and to explore potential targets for AD therapy.

Nowadays, many AD-related biomarkers have been identified with the development of high-throughput sequencing technology (7–9). However, the expression patterns and specific functions of these genes in AD still need to be further delineated. Single-cell RNA sequencing (scRNA-seq) enables gene function research under single-cell resolution. scRNA-seq technology has been widely used in the study of aortic aneurysm and dissection. A new type of AD-specific synthetic SMCs has been identified by using scRNA-seq. Further investigation showed that the synthetic SMCs are derived from the transformation of contractile SMCs and are driven by the transcription factor complex AP-1 (10). Chakraborty et al. identified a type of inflammatory SMCs by integrating scRNA-seq and an assay for transposase-accessible chromatin with high-throughput sequencing. Mechanistically, dsDNA-STING-TBK1-IRF3 signaling regulates the phenotypic transformation from contractile SMCs into inflammatory SMCs (11). Chen et al. (12) used scRNA-seq to identify a LOX high-expression fibroblast subtype that promotes AD development by communicating with SMCs.

In this study, we screened the hub genes of AD and analyzed their functions in specific cell types by integrating multiple RNA sequencing (RNA-seq) and scRNA-seq datasets. Our study provides novel references for AD pathogenesis research and the development of relevant target drugs.

## 2 Materials and methods

### 2.1 Data acquisition

**Figure 1:**
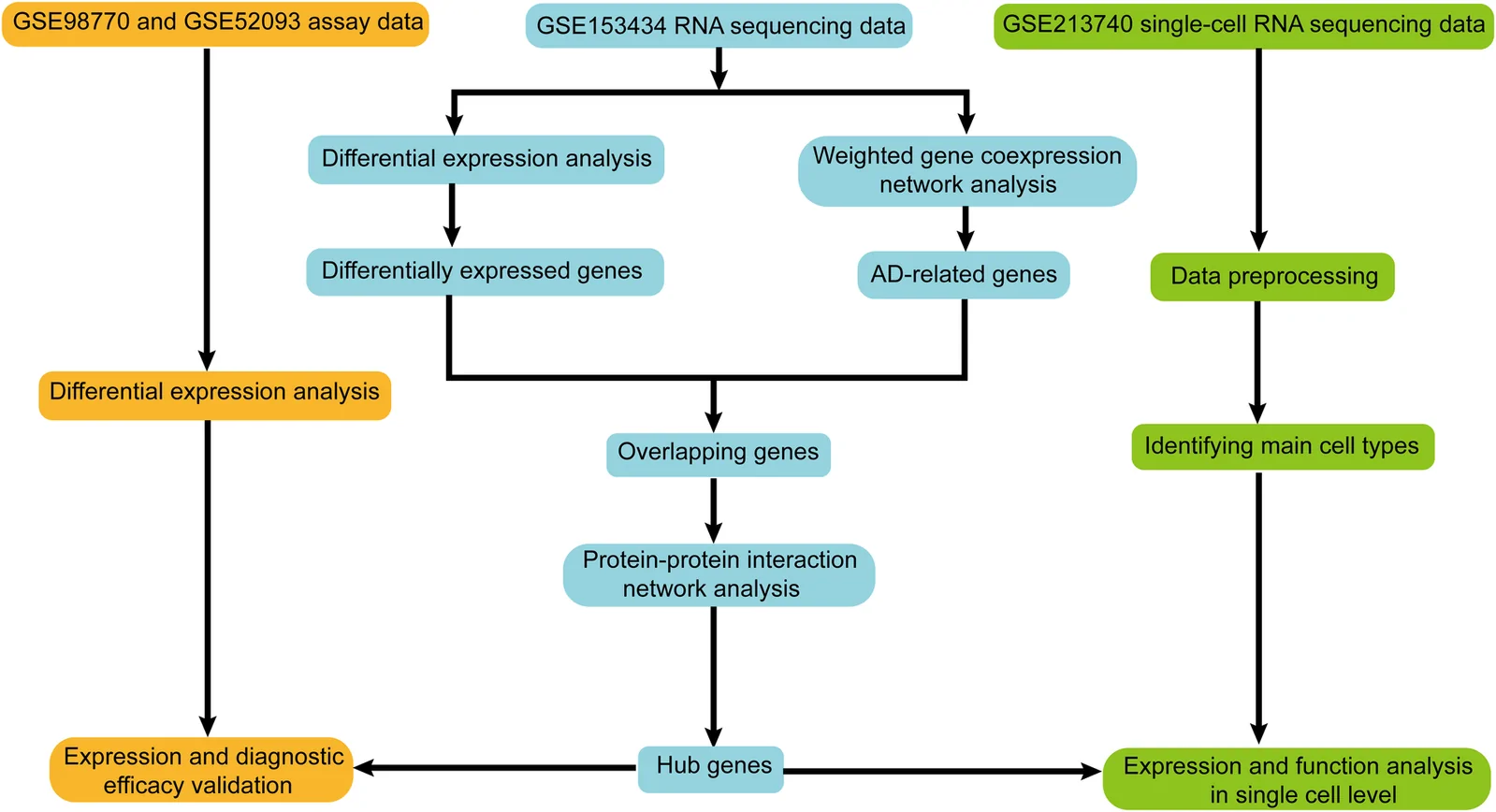
The workflow of this study. AD, aortic dissection.

Four AD-related datasets were obtained from the Gene Expression Omnibus (GEO) database (http://www.ncbi.nlm.nih.gov/geo). Table 1 displays the basic information of these datasets. The RNA-seq dataset GSE153434 containing 10 AD samples and 10 control samples was deployed to pick differentially expressed genes (DEGs) and to construct a weighted gene co-expression network to screen AD-related genes. Microarray datasets GSE98770 and GSE52093 were used to verify the expression and diagnostic efficiency of the hub genes. AD samples (*n* = 5) and control samples (*n* = 3) of scRNA-seq dataset GSE213740 were enrolled to verify the expression and function of the hub genes at the single cell level. The workflow of this study is shown in Figure 1.

**Table 1:** Datasets information used in this study.

GEO Dataset	Organism	Data type	Samples used in this study	Use of the analysis	References
GSE153434	Human	RNA-seq	10 normal, 10 diseased	WGCNA, DEGs, PPI	(13)
GSE213740	Human	scRNA-seq	5 normal, 3 diseased	Single-cell function validation	(14)
GSE98770	Human	Microarray	5 normal, 6 diseased	Hub gene expression validation and ROC analysis	(15)
GSE52093	Human	Microarray	5 normal, 7 diseased	Hub gene expression validation and ROC analysis	(16)

WGCNA, weighted gene coexpression network analysis; DEGs, differentially expressed genes; PPI, protein-protein interaction network; ROC, receiver operating characteristic curve; RNA-seq, RNA sequencing; scRNA-seq, single-cell RNA sequencing.

### 2.2 Weighted gene co-expression network analysis

**Figure 2:**
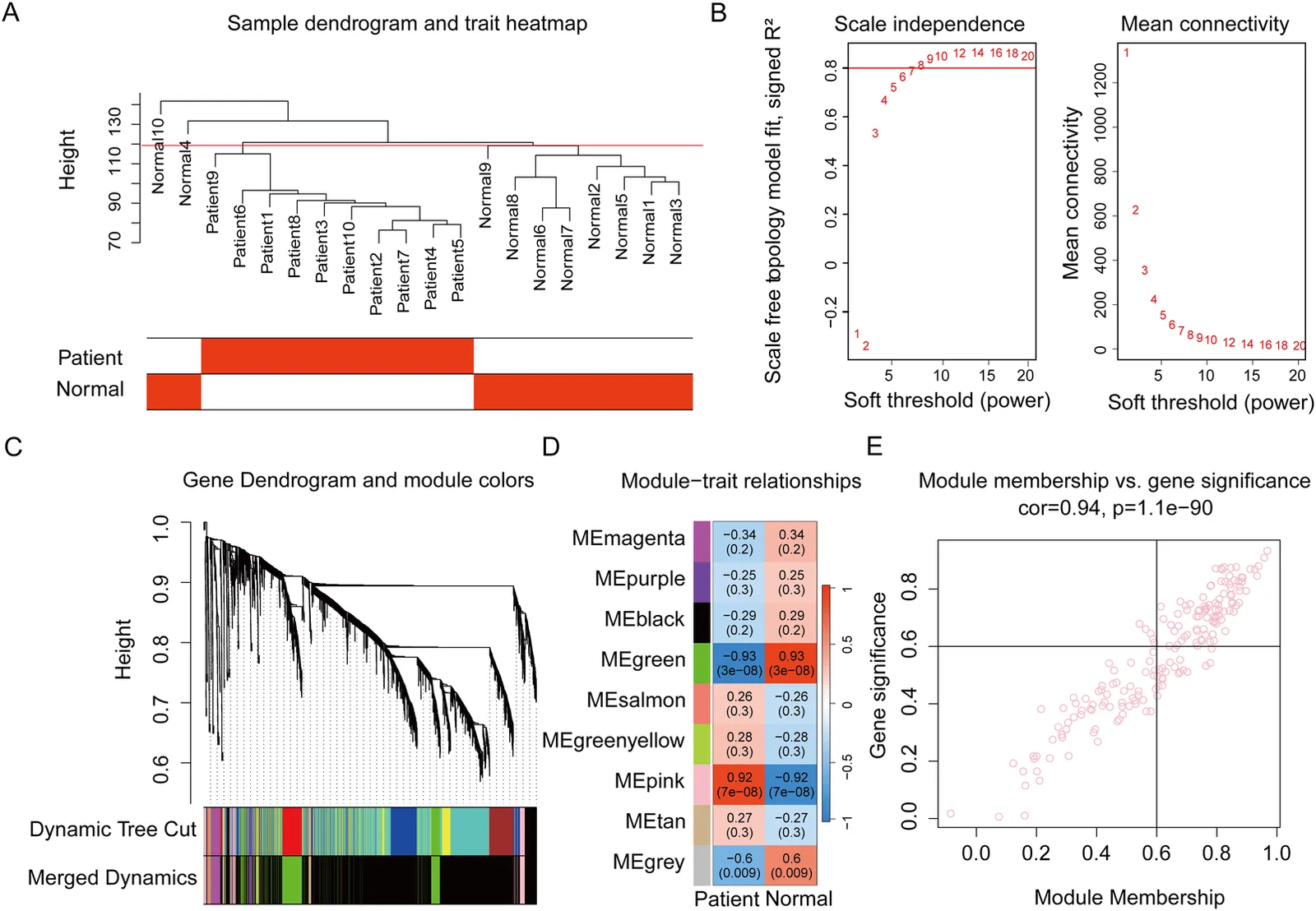
Weighted gene co-expression network analysis to identify the key gene module related to aortic dissection. **(A)** The hierarchical clustering on the samples of GSE153434. The red line shows the height of 120. **(B)** Determining the soft threshold. The red line shows the level at which *R*^2^ equals 0.8. **(C)** Gene dendrogram showing the construction of the gene co-expression modules. Fourteen modules are first identified by the cutreeDynamic function and then similar modules are merged to generate nine modules. **(D)** Module-trait relationships. Each cell shows the correlation coefficient and *P* value between the corresponding gene module and the AD patient or normal control. The pink module shows the strongest positive correlation with AD. **(E)** The module membership vs. gene trait significance plot in the pink module. AD, aortic dissection.

Hierarchical cluster analysis was performed to detect outliers in the samples derived from GSE153434. The No. 4 and No. 10 samples in the control group were removed by setting the high threshold for 120 to cut tree. Thus, we finally included 8 control and 10 patient samples in our following weighted gene co-expression network analysis (WGCNA) (Figure 2A).

The WGCNA R package (version 1.72-1) was utilized for the creation of a co-expression network and associated analyses (17, 18). First, the scale-free network topology analysis indicated that the soft threshold *β* = 8 made the *R*^2^ close to 0.8; meanwhile, the mean connectivity was below 100 (Figure 2B). Next, an adjacency matrix was calculated based on the expression matrix and transformed to the topological overlap matrix (TOM). The TOM was further transformed into the distance matrix to build a tree of gene clusters. Dynamic tree cut algorithm was used to generate different gene modules labeled in different colors. Finally, to increase the capacity of the modules, analogous modules with a cut height of <0.25 were combined.

Along with module merging, the module eigengene (ME) of each module was determined. The Pearson correlation coefficient with the sample grouping variable of each ME was calculated. Gene significance (GS) and module membership (MM) were calculated to investigate the connection between the pink module and patients. The significant genes in the pink module were selected by setting the criteria GS > 0.6 and MM > 0.6.

### 2.3 Differentially expressed gene analysis

GEO2R online tool and limma R package (version 3.58.1) were used to analyze DEGs of GSE153434, GSE98770, and GSE52093 datasets (19). Adjust *P*-value <0.05 and |log-fold change| ≥1.0 was used as the threshold criteria. A volcano plot was plotted by the ggplot2 package (version 3.4.2).

### 2.4 Venn diagram

The overlapping genes of DEGs and the significant genes in the pink module were picked by the Venn diagram generated by VennDiagram R package (version 1.7.3).

### 2.5 Protein–protein interaction network

The overlapping genes were extracted and then entered into the STRING database (https://cn.string-db.org) to construct a protein–protein interaction (PPI) network. The network type was chosen as the full string and the required score was set to 0.4 (medium confidence). Cytoscape software (version 3.7.1) was used for visualization of the network. Five methods, closeness, degree, EPC, MCC, and MNN of cytoHubba plug-in, were used to identify hub genes.

### 2.6 Receiver operating characteristic curve

The receiver operating characteristic (ROC) curve was utilized to evaluate the diagnostic efficacy of the hub genes. The pROC R package (version 1.18.4) was used to build the ROC models based on the gene expression data (20). The area under the curve (AUC) was given by the module simultaneously.

### 2.7 Single-cell RNA sequencing data analysis

Seurat R package (version 5.0.0) was performed to assess the expression of the hub genes in the scRNA-seq dataset GSE213740 (21). The Seurat object was created with the function “CreateSeuratObject” and preliminary filtering parameters “min.cells = 3, min.features = 200” were set. The mitochondrial gene expression ratio was calculated with the function “PercentageFeatureSet.” The cells were selected according to the standard of “nFeature_RNA >500 & nFeature_RNA <5,000 & percent.mt <25.” The raw counts were normalized through the “LogNormalize” method. A principal component analysis (PCA) was conducted with the function “RunPCA.” Samples were integrated with the “IntegrateLayers” function and reciprocal PCA (RPCA) method. The “ElbowPlot” function and the 1–30 dimensions were used to perform “FindNeighbors.” Then cells were clustered with the “FindClusters” function under 0.4 resolution and annotated by the canonical cell markers from the CellMarker (http://xteam.xbio.top/CellMarker/) database and literature. The uniform manifold approximation and projection (UMAP) was generated by using the “RunUMAP” function. The differential genes were calculated using the “FindMarkers” function with the Wilcox method.

### 2.8 Gene Ontology enrichment analysis

Gene Ontology (GO) enrichment analysis with over-representation analysis methods was performed by a ClusterProfiler R package (version 4.8.2) (22). Terms with adjusted *P* value <0.05 were considered significant. The bar chart was plotted by the ggplot2 package.

### 2.9 Mouse thoracic aortic aneurysm and dissection (TAAD) model

Three-week-old male C57BL/6J mice were fed a diet containing 0.4% beta-aminopropionitrile (BAPN) (Sigma-Aldrich, St. Louis, MO, USA) to establish the TAAD model as previously reported (23), and the same aged mice fed with regular diet were served as controls. We chose 10 days to represent the early stages prior to TAAD formation to detect the changes in gene expression. Mice were euthanized by intraperitoneal injection of overload sodium pentobarbital (100 mg/kg). Then the entire aortas were separated for RNA extraction.

### 2.10 Quantitative real-time polymerase chain reaction (qRT-PCR)

Total RNA was extracted by using TRIzol (Invitrogen, Carlsbad, CA, USA). Equal amounts (1 μg) were reverse-transcripted into cDNA by using HiScript III All-in-one RT SuperMix Perfect for qPCR (Vazyme, Nanjing, Jiangsu, China). ChamQ Universal SYBR qPCR Master Mix (Vazyme, Nanjing, Jiangsu, China) was used according to the manufacturer's instructions. All amplification reactions were carried out in a program involving a step at 95℃ for 30 s followed by 40 cycles of 95℃ for 10 s and 60℃ for 30 s. The mRNA expression levels were normalized to those of GAPDH using the 2^(-ΔΔCt) method. The primer sequences for the target genes are listed in Table 2.

**Table 2:** List of primers used for mouse aorta.

Gene symbol	Forward primer (5′-3′)	Reverse primer (5′-3′)
*Hif1a*	GGTTCCAGCAGACCCAGTTA	ATGCCTTAGCAGTGGTCGTT
*Plau*	ATTCCTGCAAGGGCGATTCT	AGGAAGTGTGAGACCCTCGT
*Tlr2*	CACTGGGGGTAACATCGCTT	AGTCAGGTGATGGATGTCGC
*Mif*	CTTTGTACCGTCCTCCGGTC	CGTTCGTGCCGCTAAAAGTC
*Alox5ap*	TGGCTACATCTTCGGCAAGC	ATCGTCGTGCTTACCGTTCT
*Serpine1*	CTCCAAGGGGCAACGGATAG	AAGCAAGCTGTGTCAAGGGA
*Vegfa*	GTGGGACTGGATTCGCCATT	TCCTCCCAACACAAGTCCAC
*Hgf*	CCAAACTTCTGCCGGTCCT	TCCTGATACACCTGTTGGCAC
*Met*	CCCAGCCCAAACTACCTCTG	ACCAGCTTTGGGAGGCTAAC
*Egfr*	GCAATGTTCCCATCGCTGTC	CAGGTGTCTTTGCATGTGGC
*Igf1r*	GCCTCCAACTTCGTCTTTGC	TCAATCCGTTGGGGTTCTCG
*Kdr*	GGACGAGGAGAGAGGGTCAT	ACTGGTGTGAGTGATTCGCC

### 2.11 Statistic analysis

Wilcoxon Mann–Whitney test was used to compare gene expression between two groups. *P* value <0.05 was considered to be significant. Pearson correlation coefficients were calculated to assess the correlation between genes. Genes with *P* value <0.05 and |correlation coefficient| ≥0.6 were considered to be significant.

## 3 Results

### 3.1 WGCNA to screen AD-related genes

We used WGCNA to identify the genes linked to AD pathogenesis and gene co-expression modules were built according to the process described above. The dynamic tree cut algorithm produced 14 modules. By combining the similar modules, nine modules were harvested (Figure 2C). We subsequently determined the correlation between each module and AD patients, except the gray module in which genes were not co-expressed. The results showed that the green module had the lowest negative correlation coefficient with AD (*r* = −0.93, *P* = 3e-08), while the pink module displayed the strongest positive correlation with AD (*r* = 0.92, *P* = 7e-08) (Figure 2D). The module–trait relationship was further identified by the correlation seen in the GS-MM plot in the pink module (Figure 2E). A total of 89 significant genes in the pink module were selected for further analysis.

### 3.2 Differential expression analysis in AD patients

**Figure 3:**
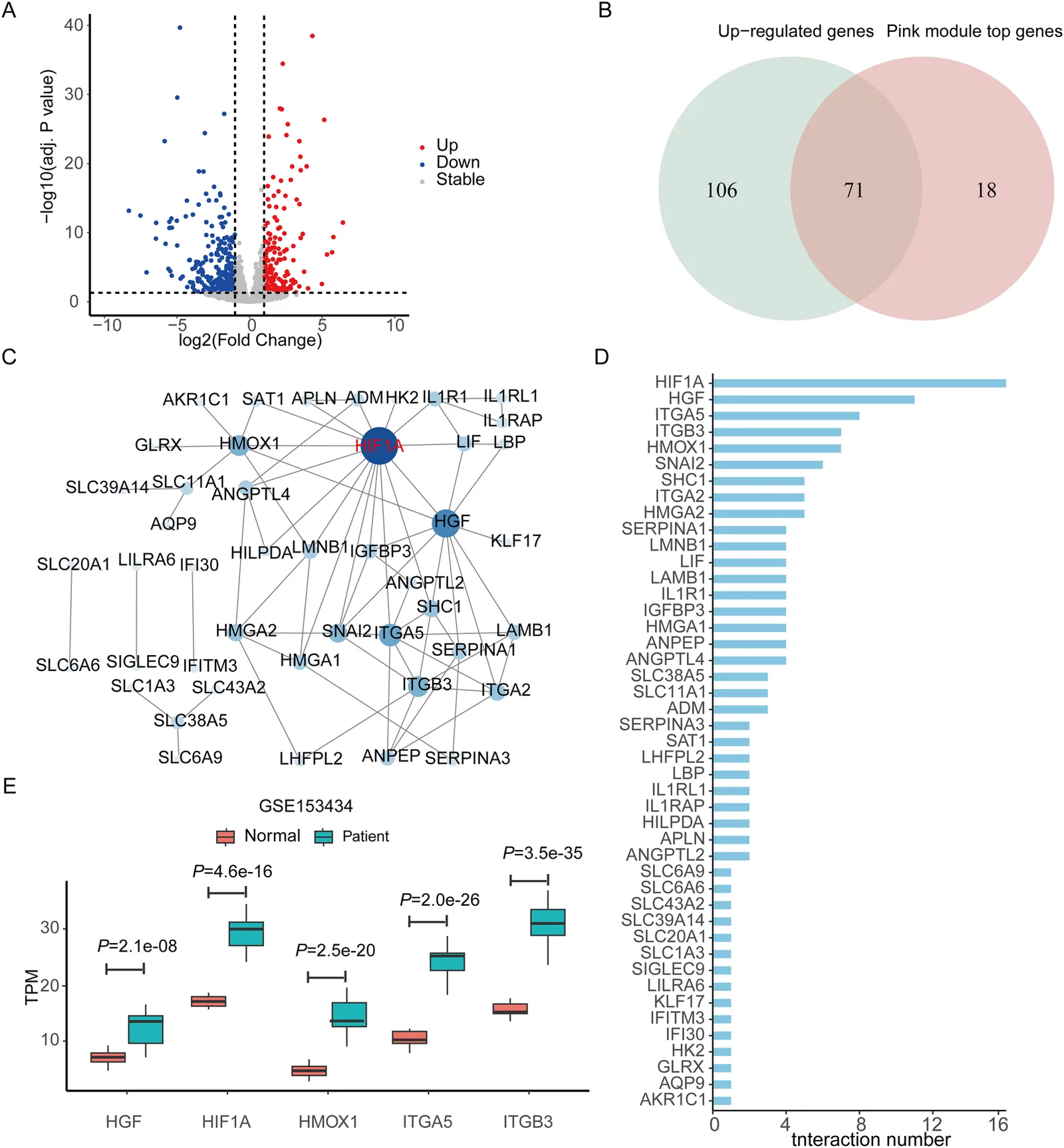
Screening differentially expressed genes and constructing protein–protein interaction network to identify the hub genes. **(A)** Volcano plot showing the DEGs of dataset GSE153434. **(B)** Venn diagram showing the overlapping genes between the DEGs and the significant genes in the pink module of WGCNA. **(C)** The PPI network is constructed based on the overlapping genes. The deeper the color, the more the number of genes interacting with them. **(D)** The interaction number of each gene in the PPI network. **(E)** The TPM value of the top five genes with the highest number of interactions in the PPI network compared between the patient and normal control groups. DEGs, differentially expressed genes. WGCNA, weighted gene co-expression network analysis. PPI, protein–protein interaction network. TPM, transcripts per kilobase of exon model per million mapped reads.

DEGs of the GSE153434 dataset were analyzed as a complement to the co-expression analysis. There were 419 DEGs, including 177 upregulated genes and 242 downregulated genes (Figure 3A). A total of 71 overlapping genes were acquired by intersecting the DEGs with the significant genes in the pink module (Figure 3B).

### 3.3 Construction of PPI network to screen hub genes in AD

All the overlapping genes were used to build the PPI network to screen the hub genes. We got a PPI network with 45 nodes and 74 edges after eliminating the genes that had no connections to others (Figure 3C). Figure 3D shows the number of the interaction genes for each gene. We used five different algorithms to calculate the hub genes respectively (Table 3). By integrating the results of the five methods, we identified the top five hub genes, including hypoxia-inducible factor 1 subunit alpha (*HIF1A*), hepatocyte growth factor (*HGF*), heme oxygenase 1 (*HMOX1*), integrin subunit alpha 5 (*ITGA5)*, and integrin subunit beta 3 (*ITGB3*).

**Table 3:** Top 10 hub genes calculated by five different methods in cytoHubba.

Rank	Closeness	Degree	EPC	MCC	MNN
1	*HIF1A*	*HIF1A*	*HIF1A*	*HIF1A*	*HIF1A*
2	*HGF*	*HGF*	*HGF*	*HGF*	*ITGA5*
3	*HMOX1*	*ITGA5*	*ITGA5*	*ITGA5*	*HGF*
4	*ITGA5*	*ITGB3*	*SNAI2*	*ITGB3*	*ITGB3*
5	*SNAI2*	*HMOX1*	*ITGB3*	*ITGA2*	*ITGA2*
6	*SHC1*	*SNAI2*	*SHC1*	*SNAI2*	*SNAI2*
7	*IGFBP3*	*HMGA2*	*IGFBP3*	*SHC1*	*HMOX1*
8	*LMNB1*	*ITGA2*	*ITGA2*	*LAMB1*	*LAMB1*
9	*LIF*	*SHC1*	*HMOX1*	*LIF*	*SHC1*
10	*HMGA1*	*IGFBP3*	*LAMB1*	*LMNB1*	*IGFBP3*

**Figure 4:**
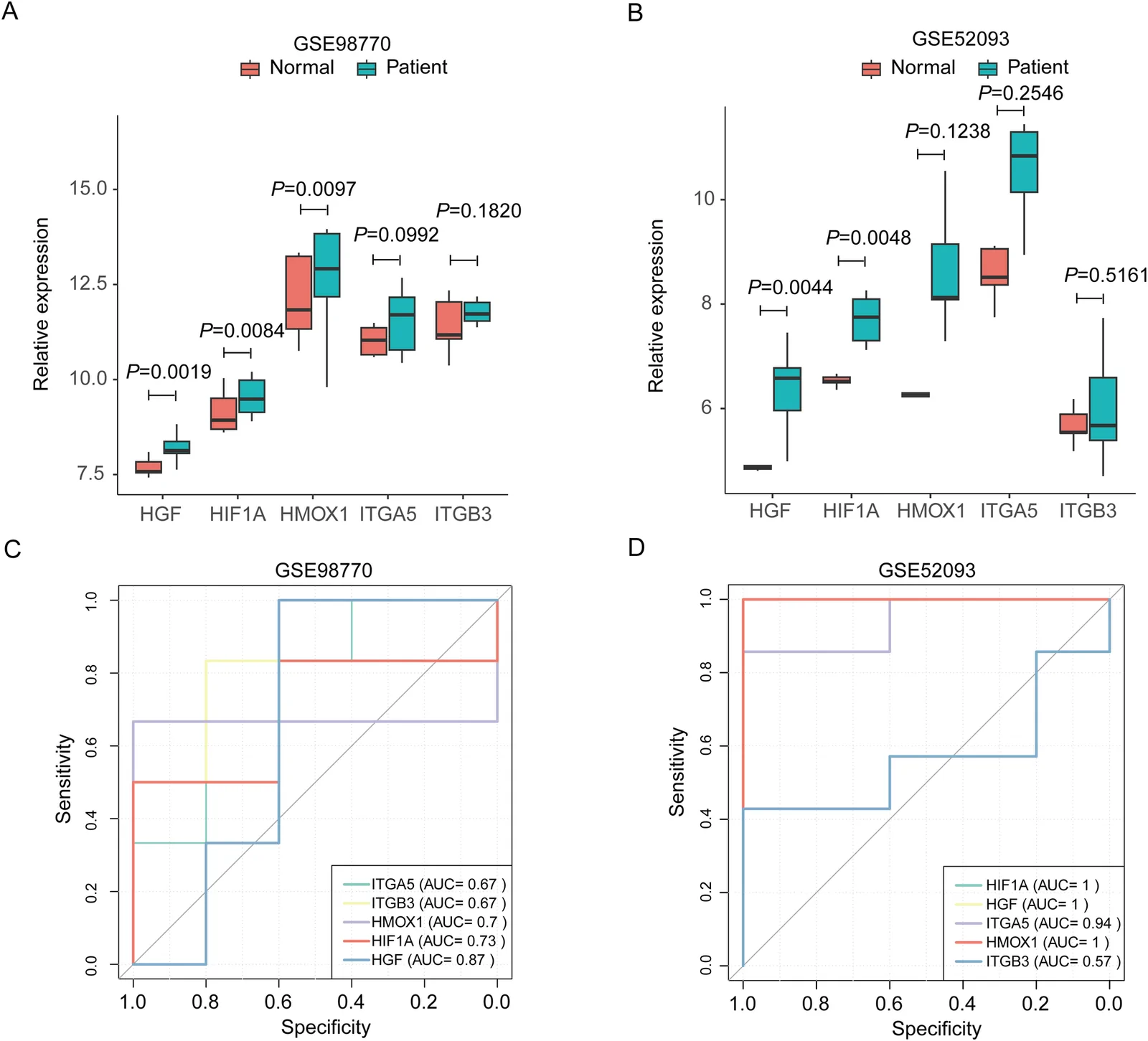
Expression and diagnostic efficiency validation of the top hub genes in extra datasets. **(A,B)** Expression validation in GSE98770 **(A)** and GSE52093 **(B–D)** diagnostic efficacy validation with receiver operating characteristic curve in GSE98770 **(C)** and GSE52093 **(D)**. *HIF1A*, hypoxia-inducible factor 1 subunit alpha. *HGF*, hepatocyte growth factor; *HMOX1*, heme oxygenase 1; *ITGA5*, integrin subunit alpha 5; *ITGB3*, integrin subunit beta 3.

We evaluated the expression and diagnostic effectiveness of the five hub genes for AD in two testing datasets GSE98770 and GSE52093. The expression of *HIF1A* and *HGF* increased significantly in the patient group compared to the control (normal) group in both datasets (Figures 4A,B). All these genes in both datasets had AUC values above 0.55, as shown by ROC curves in Figures 4C,D. These results indicate that these genes, especially *HIF1A* and *HGF*, have good diagnostic efficacy.

### 3.4 Validation of the expression pattern of hub genes in scRNA-seq data

**Figure 5:**
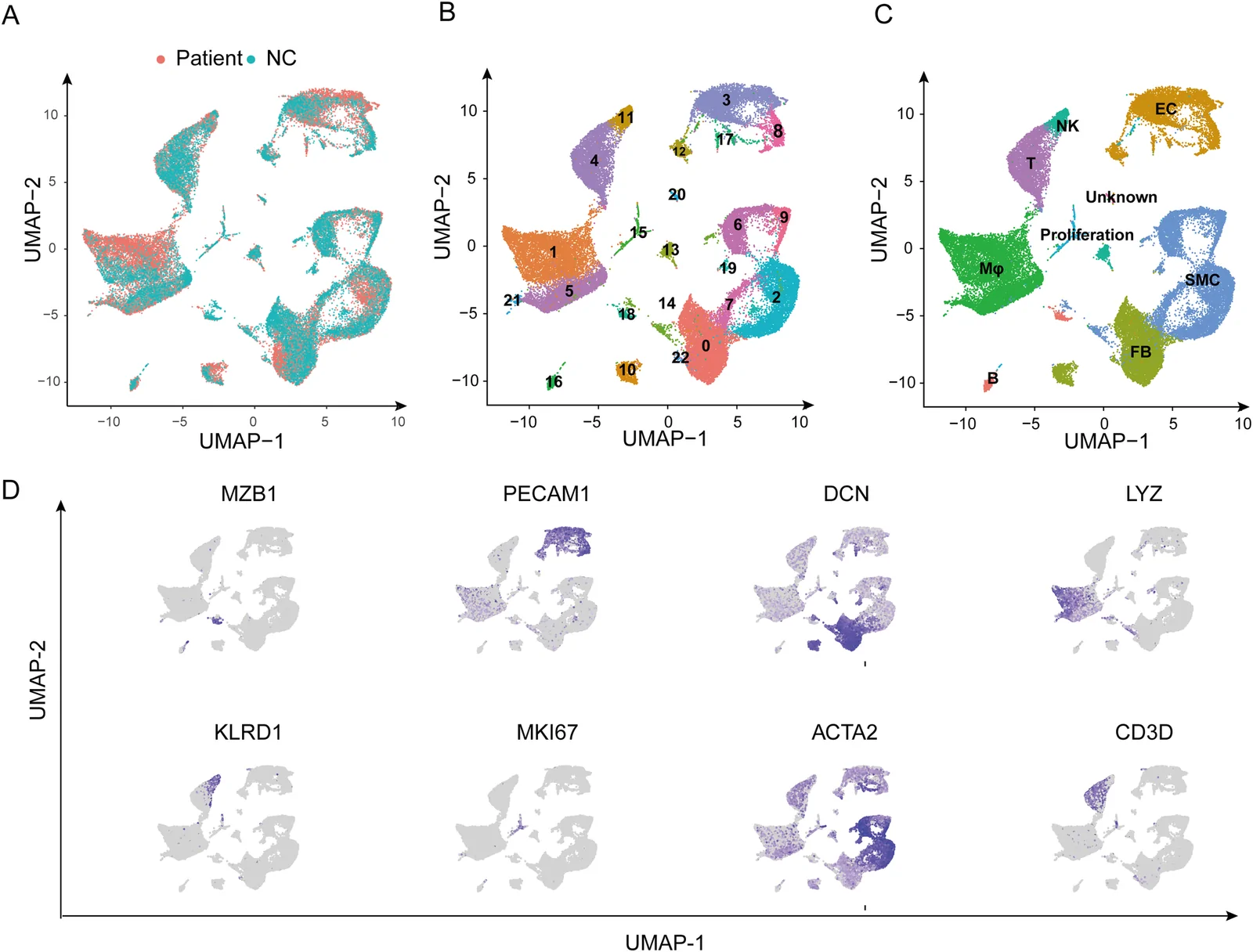
Single-cell RNA sequencing dataset GSE213740 analysis. **(A)** The UMAP plot showing that 86,131 cells from eight samples are grouped by patients and the normal controls. **(B,C)** The UMAP plot showing cells are clustered into 23 clusters and are annotated as eight main types with canonical cell markers. **(D)** Expression distribution of selected markers mapped on the UMAP plot. NC, normal control; UMAP, the uniform manifold approximation and projection; SMC, smooth muscle cell; EC, endothelial cell; FB, fibroblast; M*φ*, macrophage; T, T lymphocyte; B, B lymphocyte; NK, natural killer cell.

The scRNA-seq dataset GSE213740 was used to investigate the expression pattern of the hub genes. We first gained 23 clusters under 0.4 resolution from 8 samples and 86,131 cells (Figure 5A,B). Then each cluster was annotated with the canonical cell markers. As a result, eight main cell types, including SMCs, endothelial cells, fibroblasts, macrophages, T lymphocytes, B lymphocytes, natural killer cells, and proliferation cells, were identified (Figure 5C). Figure 5D shows the expression distribution of selected markers mapped on the UMAP plot.

**Figure 6:**
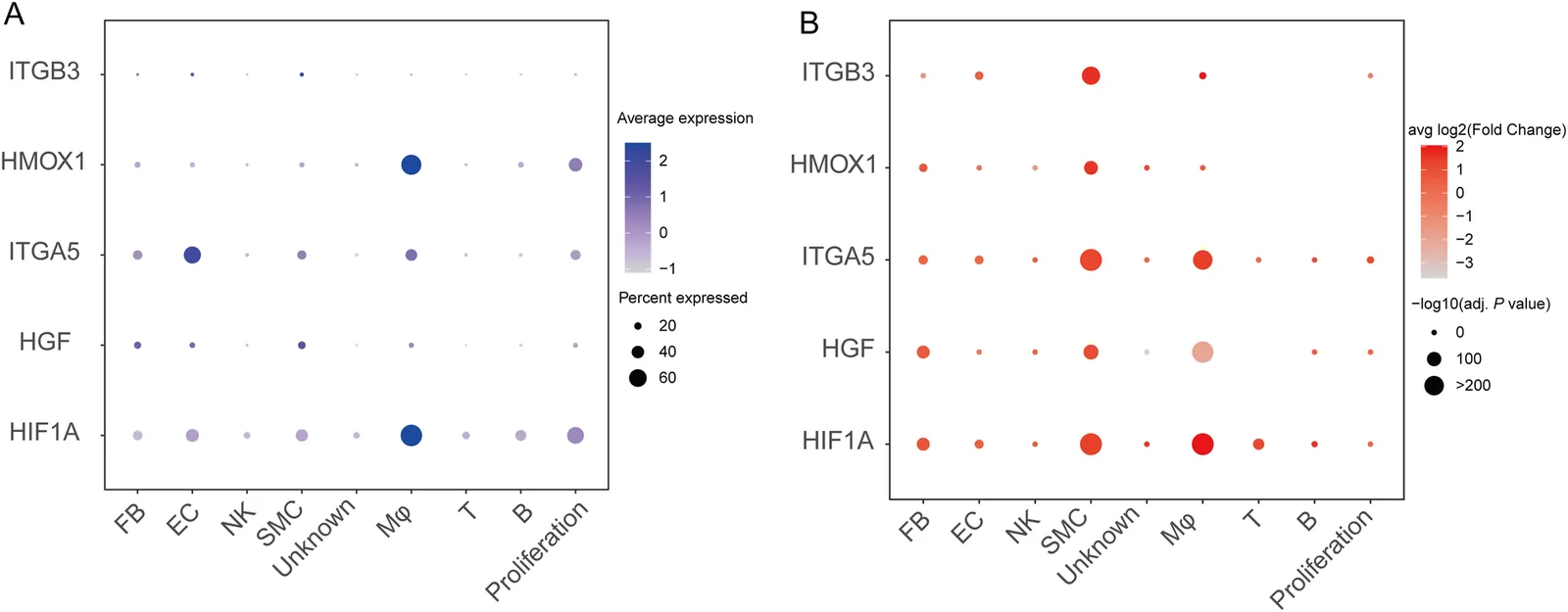
Validation of mRNA expression of the hub genes in single-cell RNA sequencing data of aortic dissection. **(A)** The dot plot shows the expression level of the hub genes in different cell types. **(B)** The dot plot shows the expression change of the hub genes in patients compared to the normal subjects. SMC, smooth muscle cell; EC, endothelial cell; FB, fibroblast; Mφ, macrophage; T, T lymphocytes; B, B lymphocytes; NK, natural killer cell.

Then we analyzed the single-cell location of five hub genes. We found that *HIF1A* was expressed in almost all the cell types with the highest expression level in macrophages. *HGF* was expressed with a certain level in SMCs, endothelial cells, and fibroblasts but with low expression in other cell types. *ITGA5* had the highest expression level in endothelial cells, followed by macrophages, SMCs, fibroblasts, and proliferation cells. *HMOX1* was highly expressed in macrophages. In contrast, the expression of *ITGB3* was quite low in all cell types (Figure 6A).

We further analyzed the expression alterations of the five hub genes in the patient group compared to the control group. We found that *HIF1A* was significantly upregulated in macrophages, SMCs, and T lymphocytes. *HGF* was significantly upregulated in SMCs. *ITGA5* was significantly upregulated in SMCs, macrophages, and proliferation cells. *HMOX1* was significantly upregulated in SMCs. *ITGB3* was significantly upregulated in SMCs and macrophages.

### 3.5 Potential function of HIF1A in AD pathogenesis

**Figure 7:**
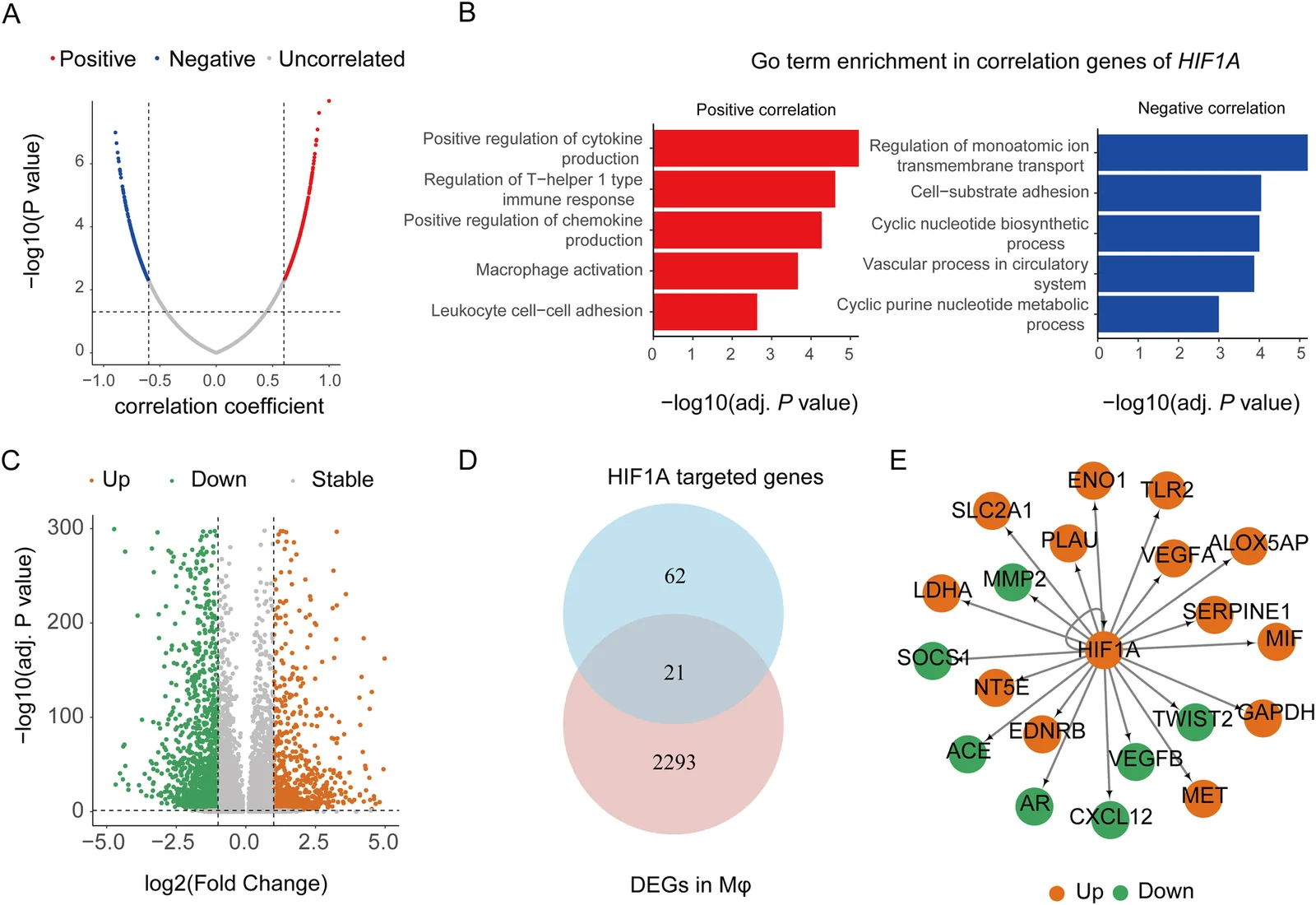
*HIF1A* function analyses of macrophages in aortic dissection. **(A)** Volcanoplot showing correlation coefficients and *P* values between *HIF1A* and other genes in GSE153434. **(B)** Representative GO terms for positively and negatively correlated genes of *HIF1A*. **(C)** DEGs of macrophages in the patient group compared to the normal group. **(D)** The number of overlapping genes between the targeted genes of *HIF1A* in the TRUST database and DEGs in macrophages. **(E)** Network plot showing the DEGs of macrophages targeted by *HIF1A*. GO, Gene Ontology; DEGs, differently expressed genes; Mφ, macrophage.

Given the high expression of *HIF1A* in macrophages and its upregulation in AD samples, its function was further analyzed. We first screened the genes related to *HIF1A* expression in GSE153434 by calculating Pearson correlation coefficients. Two hundred and forty-nine genes were positively correlated to *HIF1A* while 246 genes were negatively correlated to *HIF1A* (Figure 7A). GO enrichment analysis showed that the positively correlated genes were mainly focused on cytokine production, macrophage activation, and leukocyte cell–cell adhesion, while the negatively correlated genes were mainly focused on ion transmembrane transport, cell–substrate adhesion, and cyclic nucleotide biosynthesis (Figure 7B). Next, we analyzed the DEGs of macrophages in the patient group compared to the control group (Figure 7C). Considering that the proteins encoded by *HIF1A* are transcription factors, we determined the downstream functions that *HIF1A* may regulate directly by predicting the target genes of HIF1A using the TRUST database (https://www.grnpedia.org/trrust/). By intersecting the target genes of HIF1A and the DEGs of macrophages, we got 21 overlapping genes (Figure 7E). Among them, the upregulated genes were related to the inflammation (e.g., *TLR2*, *ALOX5AP*, and *MIF*), glycolysis (e.g., *ENO1*, *LDHA*, and *GAPDH*), tissue remodeling (e.g., *PlAU*), and angiogenesis (e.g., *SERPINE1* and *VEGFA*).

### 3.6 Potential function of HGF in AD pathogenesis

**Figure 8:**
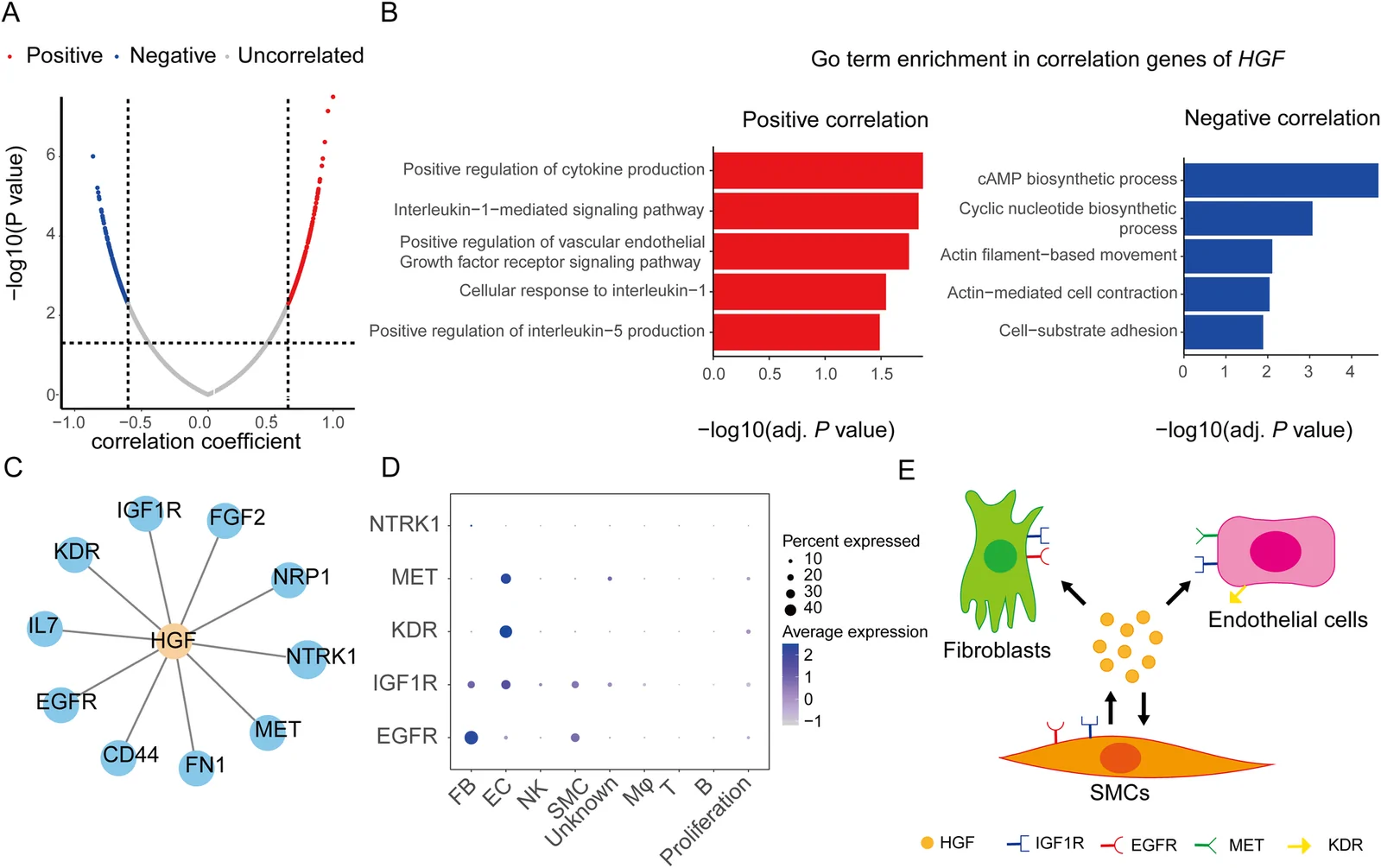
*HGF* function analysis of SMCs in aortic dissection. **(A)** Volcanoplot showing correlation coefficients and *P* values between *HGF* and other genes in GSE153434. **(B)** Representative GO terms for positively and negatively correlated genes of *HGF*. **(C)** The interaction proteins of HGF predicted through the STRING database. **(D)** mRNA expression of receptors to which HGF may interact in different cell types. **(E)** Schematic diagram showing HGF mediating the signaling between SMCs, FBs, and ECs. GO, Gene Ontology; SMC, smooth muscle cell; EC, endothelial cell; FB, fibroblast; M*φ*, macrophage; T, T lymphocytes; B, B lymphocytes; NK, natural killer cell.

We also analyzed the function of *HGF* which was highly expressed in SMCs and significantly upregulated in AD samples in dataset GSE153434. Pearson correlation coefficients showed that there were 186 and 167 genes positively and negatively correlated to *HGF*, respectively (Figure 8A). GO enrichment analysis showed that the positively correlated genes were mainly focused on cytokine production, the interleukin-1-mediated signaling pathway, and the vascular endothelial growth factor receptor signaling pathway, while the negatively correlated genes were mainly focused on the cAMP biosynthetic process, the cyclic nucleotide biosynthetic process, and actin filament-based movement (Figure 8B). Given that the proteins encoded by *HGF* are cytokines, we analyzed the receptors to which HGF may bind to. We searched for proteins that interacted with HGF in the STRING database, and 10 proteins were harvested (Figure 8C). Among them, five proteins were receptors, namely, MET, NTRK1, EGFR, IGF1R, and KDR. We assessed the gene expression level of these receptors in different cell types of the ascending aortas (Figure 8D). *MET* and *KDR* were specifically highly expressed in endothelial cells. *IGF1R* had a certain expression level in fibroblasts, endothelial cells, and SMCs. The expression of *EGFR* was highest in fibroblasts, followed by SMCs (Figure 8D). Based on the above information, we speculated that HGF secreted by SMCs may bind to their own receptors EGFR and IGF1R in an autocrine manner. In addition. HGF may also bind to EGFR and IGF1R in fibroblasts and MET, IGF1R, and KDR receptors in endothelial cells to regulate their biological functions in a paracrine manner. Thus, HGF may play an important role in the cross talk between fibroblasts, endothelial cells, and SMCs (Figure 8E).

### 3.7 Validation of mRNA expression of Hif1a and Hgf in BAPN-induced mouse TAAD model

**Figure 9:**
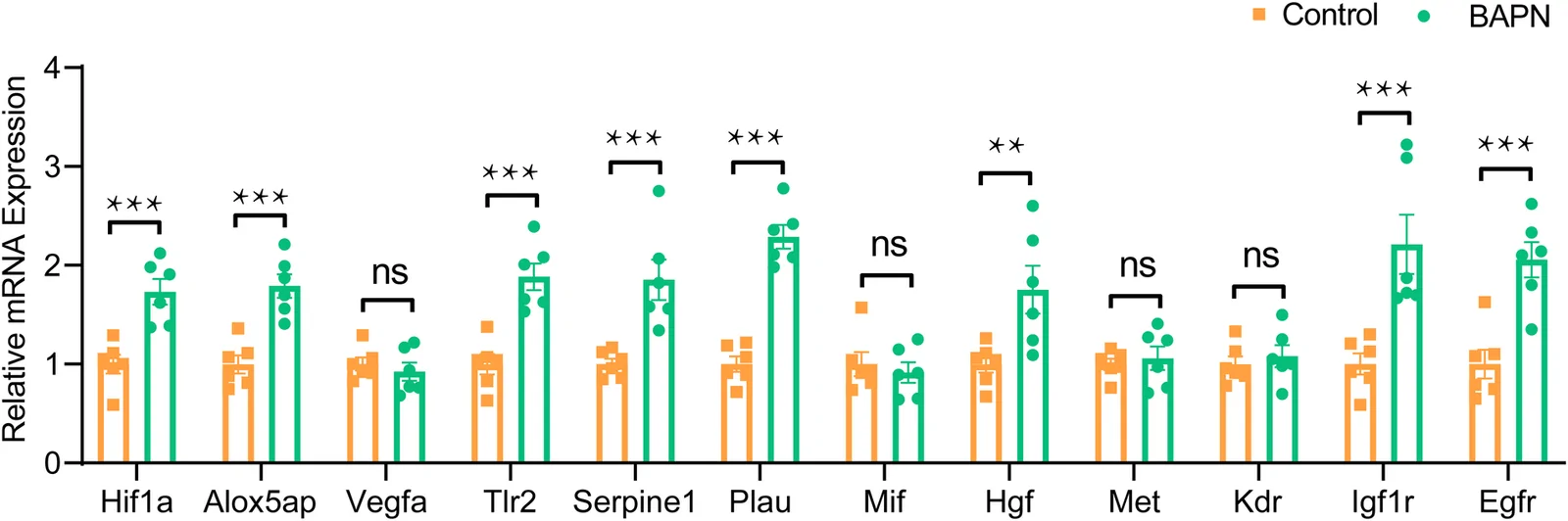
mRNA expression of *Hif1a*, *Hgf*, and their potential target genes in BAPN-induced mouse TAAD mode. Data are mea*n* ± SEM of six samples per group. BAPN, beta-aminopropionitrile. TAAD, thoracic aortic aneurysm and dissection. **P < 0.05, ***P < 0.01, ns, not significant.

To verify the activation of *HIF1A* and *HGF* signaling in AD, we analyzed the mRNA expression levels of *Hif1a*, *Hgf*, and their potential target genes in a BAPN-induced mouse TAAD model using qRT-PCR. Our results showed a significant upregulation of *Hif1a* and its several target genes, including *Alox5ap*, *Serpine1*, *Tlr2*, and *Plau*, in the aortic tissues of the BAPN-treated mice compared to the control group (Figure 9). Similarly, *Hgf* and its downstream receptor genes, namely, *Egfr* and *Igf1r*, were also significantly upregulated in BAPN-treated mice compared to controls (Figure 9). These results indicate the activation of *HIF1A* and *HGF* signaling may participate in the occurrence of AD.

## 4 Discussion

The present study obtained several main findings. First, *HIF1A*, *HGF*, *HMOX1*, *ITGA5*, and *ITGB3* were identified as the AD-related top hub genes, and they had good diagnostic efficiency for AD. Second, *HIF1A* was highly expressed in macrophages and significantly increased in AD. HIF1A may transcriptionally regulate the genes associated with inflammation, glycolysis, tissue remodeling, and angiogenesis. Third, *HGF* was highly expressed in SMCs and significantly higher in AD. HGF may mediate signaling among SMCs, endothelial cells, and fibroblasts by binding to different receptors.

Accumulating studies have shown that HIF1A plays a crucial role in the inflammation process of cardiovascular disease (CVD) including AD. HIF1A activation induced by metabolic reprogramming of macrophages triggers vascular inflammation, extracellular matrix degradation, and elastic plate breakage by increasing the expression of a disintegrin and metallopeptidase domain 17 (ADAM17) (24). Inhibiting HIF1A specifically in macrophages slows down the formation of AD in a BAPN-induced mouse model (24). Hua et al. elucidated that HIF1A participates in the inflammatory response by regulating the activity of macrophages and T-helper 17 cells by analyzing different single-cell landscapes of experimental autoimmune myocarditis model (EAM) phases. Moreover, HIF1A inhibition alleviates inflammatory cell infiltration in an EAM model (25). A recent study showed that inhibition of HIF1A expression reduces stimulator of interferon gene (STING) associated proinflammatory molecule expression of macrophages in an EAM model, indicating that STING activates proinflammatory macrophage via HIF1A and herein promotes the development of autoimmune myocarditis (26).

We found that the potential targeted genes *TLR2*, *ALOX5AP*, *MIF*, *PLAU*, *SERPINE1*, and *VEGFA* of HIF1A may be linked to the progression of AD. Previous studies have noted the importance of toll-like receptors (TLRs) in vascular inflammation. TLR2 mediates Ang (angiotensin) II-induced vascular inflammation and initiates endothelial-to-mesenchymal transition in Ang II-challenged mice (27). Damage-associated molecular patterns derived from chronic kidney disease induce vascular inflammation and the progression of atherosclerosis in a TLR-dependent manner (28). Endogenous and exogenous TLR agonists upregulate *HIF1A* by activating TLR2/TLR4 in human monocyte-derived dendritic cells (DCs) and then promote DC maturation and cytokine production (29). *ALOX5AP* encodes a kind of activation protein required for the formation of leukotriene from arachidonic acid. A study found that *ALOX5AP* gene variation is associated with interindividual differences in the risk of coronary artery disease (30). *ALOX5AP* is also associated with obesity and insulin resistance, which may make a connection between adipose tissue, inflammation, and insulin resistance (31). *MIF* is a key gene mediating atherosclerotic lesion formation. MIF promotes leukocytic infiltration and inflammation by binding to and activating the chemokine receptors CXCR2 and CXCR4. Therefore, targeting MIF may be a therapeutic strategy for CVD (32). As a serine protease, PLAU (also known as u-PA, plasminogen activator, urokinase) is closely associated with cell migration, proliferation, and tissue remodeling. Upregulating of *PLAU* significantly increases the aggressiveness and invasion of MCF-7 and thus affects metastatic-related properties of breast cancer cells (33). Chen et al. identified four aortic aneurysms (AAs)-related drug targets, namely, *BTN3A1*, *FASN*, *PSMA4*, and *PLAU*, by Mendelian randomization analyses. *PLAU* and *PSMA4* were found to strongly colocalize with AAs. This supports the notion that pharmacological inhibition of PLAU and PSMA4 may reduce AA risk (34). SERPINE1 (also known as PAI-1) is a main inhibitor of u-PA and plasminogen activator, tissue type (t-PA). PAI-1 reduces plasmin production by inhibiting the activity of these plasminogen activators, inhibits fibrin degradation, and promotes thrombus formation. A recent study showed that PAI-1 also promotes angiogenesis in the retina and is regulated by HIF2A (35). Angiogenesis has been considered destructive and plays a key role in aortic aneurysm development and rupture (36). *VEGFA* is a pivotal gene related to angiogenesis. In the glioma microenvironment, macrophages secrete VEGFA, stimulating angiogenesis and supporting glioma growth (37).

Many studies have shown that the HGF/MET pathway plays a prominent role in cardiovascular protection. The HGF/MET axis promotes the proliferation and migration of endothelial cells (38). In fibroblasts, HGF/MET prevents fibrosis by suppressing the activity of TGFB1 and Ang II (39). Previous research suggested that human atherosclerotic plaques with a low HGF level tend to be more unstable (40). However, *HGF* was identified as an aging-related hub gene of AD by a recent bioinformatics analysis (7). These findings indicated that *HGF* may play a dual role in CVD.

We found all four receptors to which HGF may combine belong to the tyrosine kinase receptor (TKR) family. These receptors have an important influence on the growth, proliferation, differentiation, and survival of cells. Yan et al. showed that *Salmonella* dysregulates the EGFR signaling pathway in the intestinal epithelium and inhibits the proliferation and differentiation of stem and progenitor cells (41). TKRs and G-protein-coupled receptors (GPCRs) participate in SMC activation. In cultured SMCs, EGFRs are transcriptionally upregulated by G-protein-coupled receptors, suggesting a GPCR-EGFR cross talk (42). IGF1Rs can mediate the aldosterone-induced vascular remodeling (43). IGF1R defect reduces the inflammation area of lung tissue and proinflammatory markers but increases resolution indicators in the bleomycin-induced lung injury mouse model (44). KDR, also known as VEGFR-2, is a member of the VEGFR family. VEGFRs are expressed either in endothelial cells or in non-endothelial cells and are widely involved in the regulation of angiogenesis (45).

Most research on the regulatory mechanisms of *HIF1A* in AD has primarily focused on its role in SMCs, with relatively little attention given to macrophages (46–49). Our study contributes to the understanding of *HIF1A* in macrophages and identifies additional potential target genes and functions of *HIF1A* in the context of AD. Previous studies on *HGF* in aortic dissection have largely been limited to its role as a biomarker (50–53). Our study is the first to analyze the expression patterns of *HGF* across different cell types within the aorta, highlighting the pivotal role of SMC-derived *HGF* in facilitating intercellular communication among aortic cells.

A limitation of our study is the lack of loss-of-function experiments on the hub genes. In addition, clinical information is needed to further explore the relationship between the hub genes and disease heterogeneity.

In conclusion, we identified two hub genes *HIF1A* and *HGF* related to AD by integrating RNA-seq and scRNA-seq datasets. *HIF1A* is likely to promote inflammation, glycolysis, tissue remodeling, and angiogenesis and *HGF* may mediate signaling among SMCs, fibroblasts, and endothelial cells. Our study provides valuable clues to detecting biomarkers, further investigating the underlying mechanism, as well as developing drugs for AD treatment.

## Data Availability

The original contributions presented in the study are included in the article/Supplementary Material, further inquiries can be directed to the corresponding author.
